# Advances in the Use of Targeted Therapies in the Management of Non–Small Cell Lung Cancer

**Published:** 2018-04-01

**Authors:** Heather Wakelee, Elizabeth S. Waxman

**Affiliations:** 1 Stanford University, Stanford Cancer Institute, Stanford, California;; 2 The University of Texas MD Anderson Cancer Center, Houston, Texas

## Abstract

Mutation-targeted therapies have had a major impact on the treatment of NSCLC, even for patients with advanced disease. Methods for detecting mutations, key clinical trials of targeted therapy, and the most common toxicities of EGFR and ALK inhibition were discussed at JADPRO Live.

Advances in tumor genetics and molecular biology created the potential to identify mutations associated with carcinogenesis and tumor progression and to develop therapies that target the mutations. Improved understanding of tumor genetics has had a major impact on the treatment of non–small cell lung cancer (NSCLC), as clinicians can offer patients a variety of active mutation-targeted therapies, even for patients with advanced disease. At JADPRO Live 2017, Heather Wakelee, MD, of Stanford University, Stanford Cancer Institute in Stanford, California, and Elizabeth S. Waxman, RN, MSN, AOCN®, ANP-BC, of The University of Texas MD Anderson Cancer Center in Houston, discussed the latest developments in the use of targeted therapies to treat NSCLC.

## TARGETS AND TARGETED THERAPIES

Multiple approaches and technologies have emerged for detecting mutations in NSCLC (Hirsch, Wynes, Gandara, & Bunn, 2010): DNA sequencing, reverse transcription–polymerase chain reaction (RT-PCR), fluorescence in situ hybridization (FISH), and immunohistochemistry (IHC).

"DNA sequencing was sort of the first strategy; this is how we initially figured out the mutations," said Dr. Wakelee. "But it’s very time consuming and very costly, so not a lot of that is being done anymore. Polymerase chain reaction tests are some of the fastest strategies, but you have to know exactly which mutation you’re looking for."

Fluorescence in situ hybridization is a good strategy when searching for break-apart or fusion mutations—when a mutation does not involve a single gene, she continued. A case in point is the *ALK* gene, which is usually fused to *EML4*. Immunohistochemistry is an older technology, but remains in widespread use as a means of identifying abnormal proteins produced by mutated genes.

Historically, the search for genetic mutations involved analysis of tumor tissue. With the recognition that tumors constantly shed DNA into circulation, researchers began to develop blood-based tests for identifying mutations in tumor genes. Known popularly as liquid biopsies, blood-based tests allow patients to avoid the inconvenience and discomfort of tissue biopsies. As the sensitivity of the tests has improved, so has clinical use of the tests.

"The vision, and actually the reality now, is that when a patient is first diagnosed with lung cancer, we can do a blood draw, and from the plasma can do this testing to find a lot of the gene mutations," said Dr. Wakelee. "I saw a patient with a new diagnosis of lung cancer on a Monday, we did a blood draw, and he returned on Thursday, at which time we already had the results. He did indeed have an EGFR mutation."

"The idea is that eventually we might be able to do serial testing instead of or perhaps in conjunction with CT [computed tomography]," she added. "Where it’s really helpful now is for patients who have developed resistance, where the tumor starts to grow and we want to find out more about why. What changed in the cancer? What changed in the tumor DNA that allowed the tumor to grow despite therapy?"

**Figure 1:**
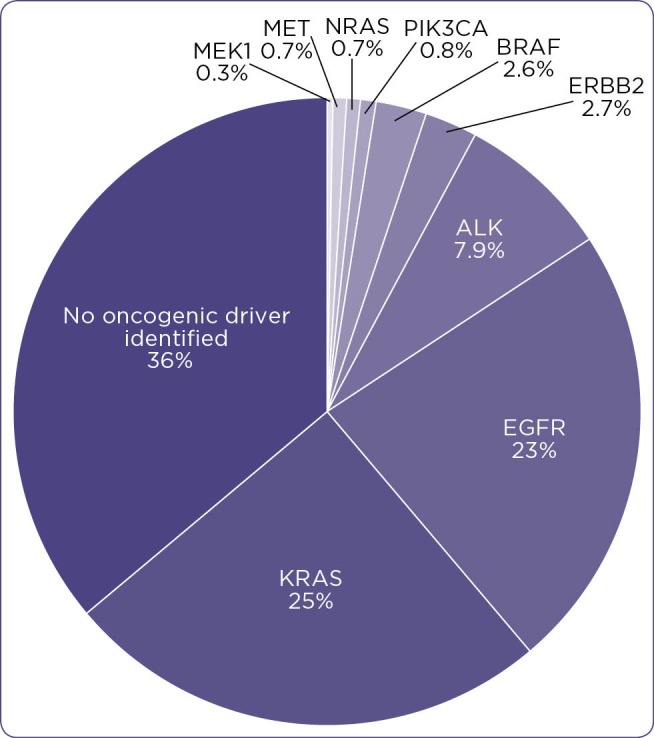
Genomic driver mutations in lung adenocarcinoma. N = 733 patients in 14 institutions of the Lung Cancer Mutation Consortium. Adapted from Sholl et al. (2015)

The search for genetic mutations in NSCLC identified multiple culprits. However, only a few mutations have proved to be important as drivers of the disease process. For example, in a study involving 733 patients with lung adenocarcinoma, the most common histologic subtype of NSCLC, driver mutations in three genes accounted for a majority of the cancers (Sholl et al., 2015): *KRAS* (25%), *EGFR* (23%), and *ALK* (7.9%). *ERBB2* (*HER2*) accounted for 2.7%, *BRAF* for 2.6%, and several others for less than 1% each. Importantly, no oncogenic driver mutation could be identified in more than a third of cases (Figure 1).

Just as different strategies are used to identify genetic mutations in NSCLC, therapies that target the mutations or mutation products employ different strategies, said Dr. Wakelee. Most therapies employ one of several strategies to target tyrosine kinases. Some target ligand binding and a resulting dimerization process necessary to activate a kinase. Others target tyrosine kinase receptors, and still others act directly on the tyrosine kinase (Noonberg & Benz, 2000).

## KEY CLINICAL TRIALS OF TARGETED THERAPY

The clinical value of targeting a mutation in NSCLC with a specific drug was demonstrated in the IRESSA Pan-Asia Study (IPASS). Early studies with EGFR tyrosine kinase–targeted drugs showed that about 10% of patients had dramatic responses to the drugs, but the reasons were unclear. Subsequently, a phenotype emerged for patients who had dramatic responses to EGFR tyrosine kinase inhibitors (TKIs): no history of smoking and often of Asian descent.

The IPASS trial was conducted at centers throughout Asia, where investigators enrolled patients with newly diagnosed NSCLC and who had never smoked (Mok et al., 2009). Patients received either gefitinib (Iressa; the first EGFR TKI) or chemotherapy. The overall results showed the patients allocated to gefitinib had a 12-month progression-free survival (PFS) of 24.9% compared with 6.7% for chemotherapy.

Tissue analysis showed that 261 (59.7%) IPASS patients had EGFR mutations and 176 did not. In the *EGFR* mutation–positive subgroup, treatment with gefitinib led to a 52% reduction in the hazard for progression or death as compared with chemotherapy (hazard ratio [HR], 0.48; 95% confidence interval [CI] = 0.36–0.64; *p* < .0001). In the *EGFR* mutation–negative group, patients were better off receiving chemotherapy, as those randomized to gefitinib had almost a 3-fold increase in the hazard for progression or death (HR, 2.86; 95% CI = 2.05–3.98; *p* < .0001).

"This emphasized to us the importance that we have to test," said Dr. Wakelee. "You can’t just assume, based on what the patient looks like, what kind of lung cancer they have; you have to test for it."

Multiple randomized trials followed IPASS, all of which limited enrollment to patients with *EGFR* mutation–positive NSCLC. The trials consistently showed significantly better PFS with an EGFR TKI vs. chemotherapy. In most cases, however, the median PFS did not exceed a year. The tumors developed resistance and resumed growing and spreading. In many instances, the tumor developed a new *EGFR* mutation, often the T790M resistance mutation (Yu et al., 2013; Table 1).

**Table 1:**
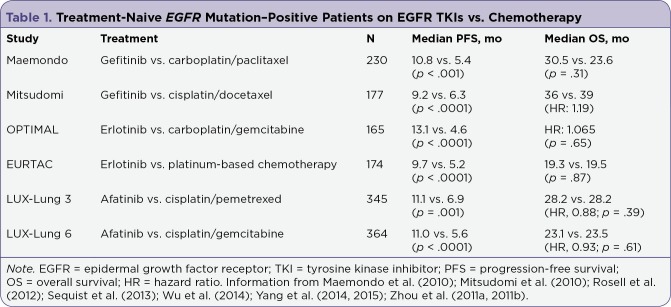
Treatment-Naive EGFR Mutation–Positive Patients on EGFR TKIs vs. Chemotherapy

"For a long time, we had nothing that worked against T790M," said Dr. Wakelee. "Several drugs were developed around the same time, but only one of them was clearly the best, and that was osimertinib."

Single-agent osimertinib (Tagrisso) demonstrated significant activity in a study involving 253 patients who had radiologic progression of NSCLC after prior EGFR TKI therapy (Jänne et al., 2015). Half of the patients had objective responses with osimertinib. In a subgroup of patients with confirmed T790M mutations, the response rate increased to 61%. In contrast, osimertinib led to an objective response rate of 21% in patients without *EGFR* mutations (Oxnard et al., 2016).

The pivotal trial of osimertinib involved 425 patients with NSCLC that progressed after first-line EGFR TKI (Mok et al., 2016). Patients were randomized 2:1 to osimertinib or chemotherapy, and the trial had a primary endpoint of PFS. Patients treated with osimertinib had more than a 2-fold increase in median PFS (10.1 vs. 4.4 months with chemotherapy). The difference represented a 70% reduction in the hazard for disease progression or death (95% CI = 0.23–0.41, *p* < .001).

Osimertinib led to objective responses in 71% of patients vs. 31% with chemotherapy, more than a 5-fold increase in the odds ratio (OR) for response (OR, 5.39; 95% CI = 3.47–8.48; *p* < .001). Grade 3 adverse events occurred half as often in the osimertinib arm of the trial.

"This actually changed practice, when the results came out," said Dr. Wakelee. "That’s why we test for the T790M mutation."

Osimertinib solidified its role as first-line therapy for *EGFR* mutation–positive NSCLC in the randomized FLAURA trial (Ramalingam et al., 2017). Patients with newly diagnosed *EGFR* mutation–positive NSCLC were randomized to osimertinib or to standard of care EGFR TKI (gefitinib or erlotinib [Tarceva]). The primary endpoint was investigator-assessed PFS, which almost doubled among patients randomized to osimertinib compared with the standard-of-care agents (18.9 vs. 10.2 months, *p* < .0001).

"The challenge is we don’t know who is going to get a T790M mutation and who is going to have a different resistance mechanism," said Dr. Wakelee. Some patients do very well by starting on erlotinib or gefitinib (on average around 10 months before progression) and then switching to osimertinib (on average around 10 months before progression) for around 20 months total time before needing to switch to chemotherapy or another strategy. However, that strategy only works well for about 50% to 60% of patients, as they are the ones who develop T790M as the resistance mechanism and thus are likely to respond to second-line osimertinib. Starting on first-line osimertinib (on average around 19 months before progression as first-line therapy) is a better strategy for most patients, although we cannot predict who will respond to second-line osimertinib and who will not. "For the 40% of people who have some other resistance mechanism, they were probably better starting on osimertinib because they’re going to have a longer PFS with that strategy," she said.

## TOXICITIES OF EGFR INHIBITION

The toxicities of EGFR inhibitors are well defined, the most common being dermatologic (rash), gastrointestinal, ophthalmic, and cardiac. The frequency and severity of toxicities vary from one agent in the drug class to another, said Ms. Waxman.

**Rash**

Upwards of 90% of patients treated with afatinib (Gilotrif) develop rash, including grade 3/4 rash in about 16%. With erlotinib, the incidence declines only slightly to 75% to 80% of patients, including 13% with grade 3/4 rash. The reported incidence of rash with gefitinib ranged from about 35% to 66%, but grade 3/4 rash was uncommon and reported in about 3% of patients. About 40% of patients treated with osimertinib develop rash, but the condition reaches grade 3/4 severity in fewer than 1% of patients.

Epidermal growth factor occurs naturally in epidermal tissue and follicular keratinocytes and has a major role in cell differentiation and protection from ultraviolet radiation and other cellular damage. Epidermal growth factor also hastens wound healing and inhibits inflammation. EGFR inhibition disrupts all those activities. The skin thins and dries out, which may result in immune system activation leading to the onset of a pustular eruption, usually accompanied by inflammation.

"The rash has a sudden onset and almost looks like acne, but it’s not because the skin is so dry," said Ms. Waxman.

The most commonly involved areas are the face, scalp, neck, upper chest, and back. Some evidence suggests that the appearance of a rash correlates with clinical benefit (Liu et al., 2013).

No standard treatment exists for anti-EGFR skin rash, said Ms. Waxman. The Multinational Association of Supportive Care in Cancer and the National Comprehensive Cancer Network have developed recommendations for managing rash, but the strategies are empirical rather than data driven. Skin care recommendations include daily skin moisturization with a thick, alcohol-free emollient; minimization of sun exposure; use of protective clothing and sunscreens rated SPF 15 or higher; lukewarm showers and baths; and avoidance of perfume- and alcohol-containing skin products (Hasenbank, 2017; Hirsh, 2011).

**Gastrointestinal Toxicity**

Diarrhea is the predominant gastrointestinal concern. Afatinib is associated with the highest incidence of diarrhea, reaching 95% in some studies, and can be dose limiting or necessitate dose reduction. Among ALK inhibitors, ceritinib (Zykadia) has the highest incidence of diarrhea, exceeding 80%, said Ms. Waxman.

Management begins with a common-sense approach to prophylaxis (Hasenbank, 2017). Patients should avoid foods that irritate the gastrointestinal mucosa: dairy, spicy foods, and greasy foods. Ongoing adequate hydration is essential. For patients treated with gefitinib or erlotinib, the onset of diarrhea is usually within the first month of treatment. With afatinib, onset can be within the first week.

After the onset of diarrhea, management consists of a bland diet (BRAT: bananas, rice, applesauce, toast), hydration and electrolyte replacement, loperamide or an alternative if loperamide is ineffective, and intravenous hydration if diarrhea reaches grade 3 severity. As a last resort, consider holding the drug or reducing the dose, said Ms. Waxman.

**Ophthalmic Issues**

Because of the presence of EGFR in and around the eye, several areas might be affected: eyelids, eyelash follicles, tear glands, conjunctiva, and cornea. Conjunctivitis occurs in about 10% of patients treated with afatinib. Patients treated with gefitinib may develop conjunctivitis, blepharitis, dry eyes, or, rarely, keratitis.

About 20% of patients treated with erlotinib report dry eyes, eyelash growth, and keratitis. A similar proportion of patients treated with osimertinib have reported dry eyes, cataracts, keratitis, blurry vision, and eye irritation.

"It’s not something to be dismissive about," said Ms. Waxman. "If your patients call you with an eye problem, have them come in." The best treatment for eye problems is referral to an ophthalmologist, she added.

## ALK INHIBITION

With EGFR inhibitors, the T790M mutation accounts for a large proportion of treatment resistance. Resistance to ALK inhibition is more complex, involving many different resistance mutations, said Dr. Wakelee. Moreover, the mutations differ according to the drug that is being used (Table 2).

**Table 2:**
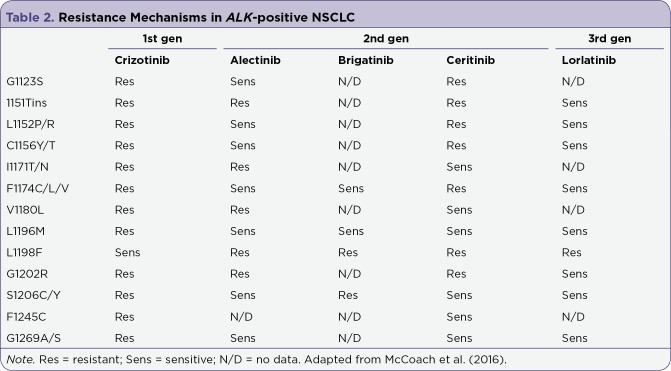
Resistance Mechanisms in *ALK*-positive NSCLC

The patient population susceptible to *ALK* resistance mutations is much broader: all races, different ages, equal distribution among men and women, smokers and nonsmokers.

Crizotinib, the first ALK inhibitor, prolonged PFS as compared with chemotherapy in a randomized trial that restricted enrollment to patients with *ALK*-positive NSCLC (Solomon et al., 2014). The results, almost a 4-month difference in PFS and a 54% reduction in the hazard for progression or death, established crizotinib as standard first-line therapy for patients with ALK-positive NSCLC.

In a relatively short time, multiple next-generation ALK inhibitors emerged for use in the second line and beyond after resistance to crizotinib occurs. Almost all of the newer ALK inhibitors are being compared against crizotinib in randomized trials that could determine whether more agents in the class should receive consideration as first-line therapy.

Results from the first of the comparative trials were reported last year and showed that initial treatment with alectinib (Alecensa) resulted in a numerically higher response rate as compared with crizotinib (83% vs. 76%, *p* = .09; Peters et al., 2017). The median PFS was 11.1 months with crizotinib but had yet to be reached with alectinib, which proved to be statistically different in favor of alectinib (HR, 0.47; 95% CI = 0.34–0.65; *p* < .0001).

"The most recent data shows that the median time to progression is 30 months with alectinib, which is quite good," said Dr. Wakelee.

Currently, three ALK TKIs have approval as first-line therapy for *ALK*-positive NSCLC: crizotinib, ceritinib, and alectinib. Ceritinib, alectinib, and brigatinib (Alunbrig) are approved for second-line therapy. Multiple other ALK inhibitors are in development, said Dr. Wakelee.

## ALK INHIBITOR TOXICITIES

Some of the common toxicities associated with ALK inhibition overlap with those observed with EGFR inhibitors: dermatologic, gastrointestinal, and ophthalmic. However, ALK inhibitors also carry a risk of cardiac toxicity, hyperglycemia, and, in the case of brigatinib, pulmonary toxicity, said Ms. Waxman.

**Vision Disturbances**

Vision disturbances are especially common with crizotinib, occurring in as many as 70% of patients. The onset of vision problems is usually within the first 2 weeks of therapies and most often involves difficulty in accommodation with light and dark. Other symptoms include shimmering or flashing lights, streamers, stringers, floaters, overlapping shadows, and afterimages.

"You don’t have to dose adjust or stop therapy; the patient can continue to take the drug," said Ms. Waxman. "I advise patients to be very careful driving or have someone drive for them. A baseline ophthalmic assessment is not necessary but might provide a measure of reassurance to the patient."

**Cardiac Toxicity**

Cardiac toxicity with crizotinib involves two principal adverse effects: sinus bradycardia and QT prolongation. In two clinical studies of crizotinib, three-fourths of patients developed bradycardia, associated with a heart rate of 50 to 59 beats per minute (bpm), and the decrease in heart rate averaged 25 bpm. The effect occurred significantly more often in patients who had a baseline heart rate of less than 70 bpm (Hasenbank, 2017; Ou, Tang, Polli, Wilner, & Schnell, 2016).

Patients with grade 1 bradycardia remain asymptomatic and do not require any medication adjustments. Grade ≥ 2 sinus bradycardia is symptomatic, and medication should be withheld until normal heart rate recovery occurs. Review all medications taken by the patient, including supplements, to determine whether any might contribute to bradycardia, said Ms. Waxman.

For grade 2 or 3 sinus bradycardia, resume the ALK inhibitor at a reduced dose if no other medications appear to be contributing to the bradycardia. If another medication is causing the slowed heart rate, adjust the medication dose and resume the ALK inhibitor at the full dose. For patients with grade 4 sinus bradycardia, the ALK inhibitor should be permanently discontinued unless another medication is determined to be the cause of the bradycardia (Ou et al., 2016).

Labeling for crizotinib, ceritinib, and osimertinib includes a boxed warning related to QT prolongation. A patient’s medical history and medications should be reviewed in advance and again if QT prolongation occurs. Baseline and follow-up electrocardiogram should be performed (Hasenbank, 2017).

**Hyperglycemia**

Normally uncommon with TKIs, hyperglycemia is a potential adverse effect of ceritinib and alectinib, owing to the drugs’ inhibition of the insulin-like growth factor receptor. Patients on diabetes medications should continue the medications, and their blood glucose level should be monitored regularly, said Ms. Waxman. If a patient’s blood glucose level rises to 180 mg/dL or higher, stop the ALK inhibitor until the glucose level returns to a safe level, then resume the inhibitor at a lower dose.

**Pulmonary Toxicity**

Limited to brigatinib, pulmonary toxicity occurs in the form of interstitial lung disease (ILD). Whenever ILD is suspected, refer the patient for a biopsy and pulmonary consultation for clinical management, if indicated. If ILD is confirmed, the ALK inhibitor should be stopped. Whether brigatinib should be resumed depends on the severity of the pulmonary reaction. Additionally, clinicians and patients now have multiple options in the ALK inhibitor category.

"Since many toxicities are unique to the different agents, we have flexibility with our patients, where if they do get one of these specific toxicities, we have the option now to switch to a different agent," said Dr. Wakelee.

## OTHER DRIVER MUTATIONS

Accounting for less than 3% of mutant NSCLC, and less than 1% in a number of instances, the remaining driver mutations nonetheless warrant clinical consideration and attention because many of them have proven susceptible to currently available drugs, Dr. Wakelee noted. For example, the *BRAF* V600E mutation—better known as a driver in melanoma—accounts for 2.6% of mutant NSCLC. However, the combination of two available drugs—the BRAF inhibitor dabrafenib (Tafinlar) and MEK inhibitor trametinib (Mekinist) resulted in an objective response rate of 63% in a phase II clinical trial involving 57 patients with *BRAF* V600E-mutated NSCLC (Planchard et al., 2016). The combination was generally well tolerated.

Cabozantinib (Cometriq), a multitargeted TKI, has approval for thyroid cancer but also has demonstrated activity in lung adenocarcinomas associated with RET rearrangement. In one small clinical trial, 7 of 25 patients had partial responses with cabozantinib (Drilon et al., 2016). Multiple small clinical trials of RET inhibitors have demonstrated activity in as many as half of patients with RET rearrangement.

Treatment with cabozantinib led to dramatic responses in a small trial of patients with NSCLC associated with *MET* exon 14 splice variant, which occurs in about 4% of lung adenocarcinomas (Paik et al., 2015). The results showed 8 confirmed responses in 18 patients and an additional 5 unconfirmed responses. Crizotinib also has MET activity and is also active in this patient population.

Most commonly found in association with breast cancer, *HER2* mutations also occur in lung cancer. High response rates have been observed with both trastuzumab plus chemotherapy and with HER2-targeted therapy such as afatinib (a dual EGFR/HER2 inhibitor; Mazières et al., 2013).
